# Gene expression profiling to elucidate the promotive effects of the volatile organic compound 3-octanone on the mycelial growth of *Ganoderma lucidum*

**DOI:** 10.1007/s10529-026-03698-5

**Published:** 2026-02-05

**Authors:** Shoko Horikawa, Ryuka Iizuka, Kiwamu Umezawa, Rumi Konuma, Makoto Yoshida

**Affiliations:** 1 United Graduate School of Agricultural Science, Tokyo University of Agriculture and Technology, Fuchu, Tokyo, 183-8509 Japan; 2 Institute of Global Innovation Research, Tokyo University of Agriculture and Technology, Fuchu, Tokyo, 183-8509 Japan; 3 Department of Applied Biological Chemistry, Faculty of Agriculture, Kindai University, 3327-204 Naka-machi, Nara, 631-8505 Japan; 4 Tokyo Metropolitan Industrial Technology Research Institute, 1-9 Kandasakuma-cho, Chiyoda-ku, Tokyo, 101-0025 Japan; 5 Institute of Agriculture, Tokyo University of Agriculture and Technology, Fuchu, Tokyo, 183-8509 Japan

**Keywords:** FVOCs, RNA-seq, Transcriptome, Wood-rotting fungi

## Abstract

To investigate the effects of fungal volatile organic compounds (FVOCs) on the mycelial growth of *Ganoderma lucidum*, and to elucidate the molecular mechanisms underlying the growth-promoting effect of 3-octanone. *G. lucidum* was cultivated with 1-octen-3-ol, 3-octanol and 3-octanone for 7 days, after which colony diameter and mycelial dry weight were measured to assess their effects on mycelial growth. RNA-seq was used to investigate gene expression changes following 3-octanone exposure. While 1-octen-3-ol or 3-octanol inhibited mycelial growth in *G. lucidum*, 3-octanone promoted it. In total, 590 differentially expressed genes (DEGs), including 162 upregulated and 428 downregulated genes, were identified following 3-octanone exposure. Functional annotation revealed that among the DEGs, 23 genes were related to fungal cell wall biosynthesis and remodeling, whereas 21 genes were involved in plant-derived polysaccharide degradation. Furthermore, significant expression changes were observed in genes related to secondary metabolism. Our results indicate that *G. lucidum* can use 3-octanone as a signal to recognize other fungi, potentially facilitating the expansion of its own territory within wood in nature.

**Supplementary Information:**

The online version contains supplementary material available at 10.1007/s10529-026-03698-5.

## Introduction

Volatile organic compounds (VOCs) are low-molecular-weight compounds that easily evaporate at normal temperature and pressure. Fungal VOCs (fungal volatile organic compounds; FVOCs) include alcohols, aldehydes, ketones, esters, terpenes, and their derivatives (Korpi et al. 2009; Inamdar et al. 2020). The production profiles of FVOCs vary by fungal species (Korpi et al. 1998; Ewen et al. 2004; Matysik et al. 2009) and growth stage (Nilsson et al. 1996; Bjurman et al. 1997; Stoppacher et al. 2010), as well as by environmental conditions such as substrate composition (Whatley et al. 1997; Wilkins et al. 2000). Some FVOCs possess biological activity, affecting spore germination, mycelial growth, and secondary metabolite production (El Jaddaoui et al. 2023). Eight-carbon compounds are recognized as the major FVOCs (Combat et al. 2006; Inamdar et al. 2020). Among them, 1-octen-3-ol, one of the most abundant FVOCs in numerous fungi, has been reported to inhibit spore germination and mycelial growth in fungi (Chittra et al. 2004; Herrera et al. 2015; Singh et al. 2020). These findings highlight the ecological roles of FVOCs, suggesting their use as communication signals between organisms (Bennett et al. 2012).

Wood-rotting fungi decompose wood in forest ecosystems through their ability to degrade wood cell walls. Wood-rotting fungi also produce FVOCs during their growth, with FVOCs production profiles differing between fungi grown on agar medium and those grown on wood substrates. In addition, FVOCs production profile was also found to change during the decay process (Konuma et al. 2015). In nature, different species of wood-rotting fungi coexist on the same tree (Boddy 2000), and they interact both before and after mycelial contact (Heilmann-Clausen et al. 2015), suggesting that FVOCs mediate biological interactions at a distance (Boddy 2000). Supporting this hypothesis, Evans et al. (2008) reported that FVOCs production profiles differed when different species of wood-rotting fungi were cultured in confrontation compared with when each species was cultured alone, suggesting the existence of FVOCs-mediated interactions between wood-rotting fungi.

Most studies on the effects of FVOCs on the growth of wood-rotting fungi have examined morphological traits, whereas the underlying molecular mechanisms remain largely unknown. In the present study, we investigated these mechanisms in the fungus *Ganoderma lucidum* (Basidiomycota), a well-studied wood-rotting fungus that is widely distributed in subtropical and temperate regions of the world, including Asia. We specifically examined the effects of the major eight-carbon FVOCs 1-octen-3-ol, 3-octanol, and 3-octanone on mycelial growth in *G. lucidum*. We subsequently performed RNA-seq analysis to investigate gene expression changes in *G. lucidum* exposure to 3-octanone.

## Materials and methods

### Fungal strains and chemicals

*G. lucidum* (NBRC 109388) was maintained on potato dextrose agar (PDA, Shimadzu Diagnostics Corporation, Tokyo, Japan) at 25 °C in the dark until use in experiments. 1-Octen-3-ol (> 98%) was purchased from Tokyo Chemical Industry (Tokyo, Japan). 3-Octanol (99%) and 3-octanone (≥ 98%) were purchased from Sigma-Aldrich (St. Louis, MO, USA).

### Effects of eight-carbon compounds on mycelial growth

Plastic Petri dishes (Φ90 mm) were prepared with different concentrations of PDA, including a normal PDA condition (39 g PDA l^−1^), 1/2 diluted PDA condition (19.5 g PDA l^−1^), and 1/4 diluted PDA condition (9.75 g PDA l^−1^). In the diluted PDA media, agar was supplemented to the same concentration as in the normal PDA medium. A mycelial plug of *G. lucidum* (Φ7 mm) was placed on the center of PDA, and a cover glass was placed inside the lid. One microliter of either 1-octen-3-ol, 3-octanol, or 3-octanone was added to the cover glass. The Petri dishes were sealed with Parafilm and cultivated at 25 °C in the dark with the medium placed on top to expose the fungus to the volatilized compounds. After 7 days, the colony diameter and mycelial dry weight were measured. Colony diameter was determined by drawing two perpendicular lines through the center of the colony and measuring the length of each line. The average of the two measurements was taken as the colony diameter, and the relative colony diameter was calculated relative to the colony diameter on control plates, which were not exposed to FVOCs (Eq. 1). To determine the mycelial dry weight, the mycelia were collected and subjected to hot water treatment at 95 °C for 30 min to dissolve the medium. The mycelia were then collected on filter paper via suction filtration and dried at 105 °C for 12 h. The weight of the filter paper containing the mycelia was measured, and the weight of the filter paper was subtracted to determine the mycelial weight. The relative mycelial weight was calculated relative to the mycelial weight of control plates (Eq. 2). Three independent experiments were performed, each with five replicates per treatment group. Dunnett’s test was used to determine the statistical significance of differences between the FVOCs-exposed and FVOCs-unexposed (control) groups.

1 $$Relative\,\, colony \,\,diameter \left( \% \right) = \frac{{Colony\,\,diameter\,\, in\,\,the\,\,presence \,\,of\,\,FVOCs\,\,\left( {mm} \right)}}{{Colony\,\,diameter\,\, in\,\,the\,\,absence\,\,of\,\, FVOCs \,\,\left( {mm} \right)}} \times 100$$

2 $$Relative\,\,mycelial\,\,weight \left( \% \right) = \frac{Dry\,\,weight\,\,of\,\,mycelia\,\,grown\,\,in\,\,the\,\,presence\,\,of\,\,FVOCs\,\,(g)}{{Dry\,\,weight \,\,of\,\,mycelia \,\,grown\,\, in\,\,the\,\,absence\,\,of\,\, FVOCs\,\,(g)}} \times 100$$

### RNA extraction and RNA-seq

*G. lucidum* was cultivated on 1/4 diluted PDA plates in the dark as described above. On day 4, a cover glass was placed inside the lid of each Petri dish. For the treatment group, 1 μL of 3-octanone was added to the cover glass, whereas no compound was added to the control group. After 2 additional days of cultivation, a section of the colony located 5 mm inward from the colony edge was collected. Four biological replicates were prepared for each group, and the samples were immediately frozen and stored at − 80 °C. Total RNA was extracted from the frozen samples using the RNeasy Plant Mini Kit (Qiagen, Hilden, Germany) and treated with DNase I (Qiagen). Strand-specific cDNA libraries for RNA-seq were prepared using the Illumina Strand mRNA Prep Ligation Kit (Illumina, San Diego, CA, USA). These libraries were sequenced a 2 × 150 bp using a NovaSeq 6000 (Illumina). cDNA library construction and subsequent RNA-seq were performed as a custom service by Genome-Lead (Kagawa, Japan).

### Bioinformatic analysis

The quality of raw reads was evaluated using FastQC v0.11.9 (https://www.bioinformatics.babraham.ac.uk/projects/fastqc/). Reads were then processed using Trimmomatic v0.39 (Bolger et al. 2014) to remove adapters and discard reads with an average quality lower than 15 or a final length shorter than 50 bp. The quality of the processed reads was re-assessed using FastQC. The processed reads were mapped to the *G. lucidum* reference genome (Chen et al. 2012) obtained from the Joint Genome Institute (JGI) MycoCosm portal (Grigoriev et al. 2012, 2014) using Hisat2 v2.2.1 (Kim et al. 2015, 2019). The resulting SAM files were converted to sorted BAM files using Samtools v1.14 (Li et al. 2009). Gene expression was then quantified from the sorted alignment files using featureCounts in Subread v2.1.1 (Liao et al. 2013, 2014) based on the gene model annotation file from JGI. Differential expression was analyzed using the edgeR method (Robinson et al. 2010) implemented through scripts from Trinity v2.15.2 (Haas et al. 2013), which includes normalization of raw counts via the trimmed mean of M-values. Differentially expressed genes (DEGs) were defined as genes with false discovery rate (FDR) < 0.05 and absolute log_2_ fold change (logFC) >|1| (equivalent to a twofold change).

Functional annotations for the predicted proteins were obtained from multiple sources. First, pre-computed annotations, including Gene Ontology (GO) terms, InterPro domains, and euKaryotic Orthologous Groups classifications, were retrieved from JGI for the *G. lucidum* genome. To further annotate the proteins, a homology search was conducted using BLASTp (the E-value cutoff was set to 1e − 5) against the Swiss-Prot database using BLAST + v2.12.0 (Camacho et al. 2009). Additionally, Carbohydrate-Active enZymes (CAZymes) were identified by submitting the protein sequences to the dbCAN3 server (Yin et al. 2012; Zheng et al. 2023), and annotations were derived from the combined HMMER (Eddy 2011) and DIAMOND (Buchfink et al. 2015) tool outputs.

To identify overrepresented GO terms within the DEGs, GO enrichment analysis was performed. The analysis was conducted separately for up- and down-regulated gene sets using the enricher function from the R package clusterProfiler v4.10.1 (Wu et al. 2021). GO terms with *p*-value < 0.05 and *q*-value (FDR) < 0.1 were considered significantly enriched.

## Results and Discussion

### Effects of FVOCs on the mycelial growth of G. lucidum

The changes in the colony diameter and mycelial dry weight of *G. lucidum* were determined to assess the effects of FVOCs exposure. To consider the effect of carbon source concentration, three different concentrations of PDA medium were used in the experiment. Table 1 presents the changes in mycelial growth associated with FVOCs exposure and with changes in carbon availability. In cultures exposed to 1-octen-3-ol, the colony diameter and mycelial dry weight were lower than the control values at all PDA concentrations (*p* < 0.01). The inhibitory effects of 1-octen-3-ol on mycelial growth have been widely reported in ascomycetes (Inamdar et al. 2020). These results suggest that 1-octen-3-ol also inhibits mycelial growth in basidiomycetes. Conversely, 3-octanol exposure did not lead to significant differences in colony diameter, and the mycelial weight was decreased only in 1/2 diluted PDA medium (*p* < 0.05). In cultures exposed to 3-octanone, the colony diameter was increased at all PDA concentrations (normal PDA medium: *p* < 0.05, 1/2 and 1/4 diluted PDA medium: *p* < 0.01). Although no significant difference in mycelial weight was observed in cultures grown on normal or 1/2 diluted PDA medium compared with the control, a significant increase was observed in 1/4 diluted PDA medium (*p* < 0.01). Previous studies on FVOCs and mycelial growth have focused on their inhibitory effects, which are considered a defense mechanism against competitive species. The finding that 3-octanone promoted mycelial growth suggests that FVOCs can also function as signaling molecules that promote growth in response to environmental changes. The promotive effect of 3-octanone on mycelial growth was more noticeable under low PDA concentrations, i.e., limited carbon source availability. The amount of 3-octanone added (1 μL) corresponded to approximately 1.5 mg of glucose (glucose concentration of approximately 0.075% in the culture). Given that such a small amount of carbon is unlikely to serve as a nutrient source to promote mycelial growth, we hypothesized that 3-octanone functions as a signaling molecule that stimulates pathways related to mycelial growth.

**Table 1:** Effects of 1-octen-3-ol, 3-octanol, and 3-octanone on the colony diameter and mycelial dry weight of *G. lucidum*

PDA concentration			Relative growth (%)		
			1-octen-3-ol	3-octanol	3-octanone
Normal	39 g l^−1^	Diameter	68 ± 3^**^	102 ± 3	113 ± 4^*^
		Weight	63 ± 3^**^	109 ± 4	118 ± 6
1/2 diluted	19.5 g l^−1^	Diameter	64 ± 2^**^	100 ± 4	124 ± 4^**^
		Weight	55 ± 6^**^	80 ± 6^*^	112 ± 5
1/4 diluted	9.75 g l^−1^	Diameter	70 ± 2^**^	105 ± 8	137 ± 4^**^
		Weight	58 ± 4^**^	94 ± 7	146 ± 8^**^

^*^Error bars represent standard errors, and asterisks indicate significant differences compared with the control at each PDA concentration (*p < 0.05, **p < 0.01). The raw values corresponding to the relative values are shown in Table S1

### Differential gene expression in response to 3-octanone

**Fig. 1:**
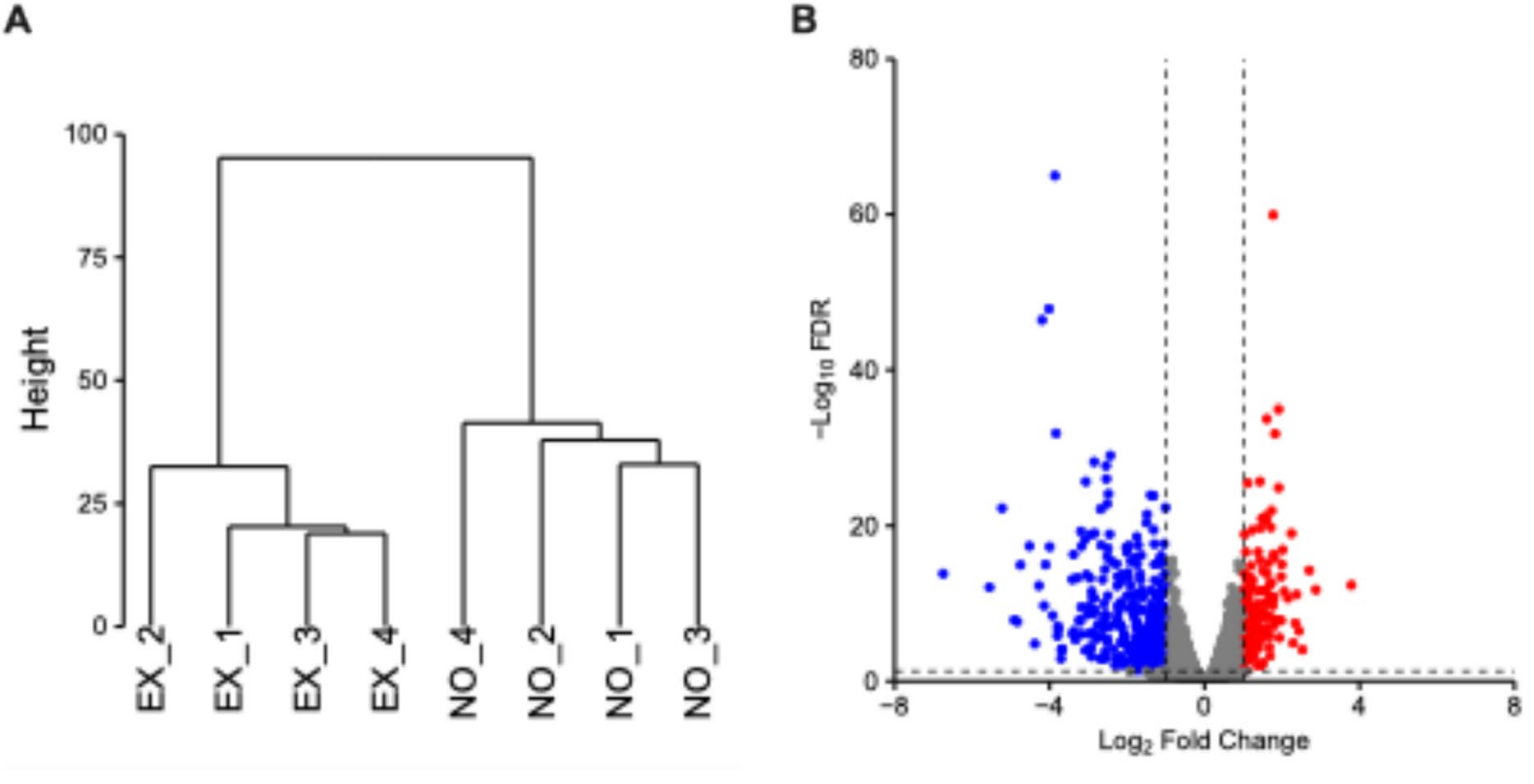
Cluster dendrogram **A** and volcano plot **B** of RNA-seq data from *G. lucidum* cultures exposed (EX) and not exposed (NO) to 3-octanone. The dendrogram was generated based on TMM-normalized gene expression profiles (log2-CPM) using Euclidean distance and Ward's linkage method. The robust clustering of replicates within each group reflects the consistency of the treatment effects and highlights a significant shift in the metabolic or physiological state. In the volcano plot, the x-axis represents the log_2_ fold change in gene expression between EX and NO groups, and the y-axis represents the -log_10_ FDR

We used comparative transcriptomic analysis to clarify the mechanism by which 3-octanone promotes mycelial growth in *G. lucidum*. Total RNA was extracted from *G. lucidum* cultured in 1/4 diluted PDA medium, where the growth-promoting effects of 3-octanone were noticeable, and was used for next-generation sequencing. Eight libraries were sequenced, yielding 25.7–49.4 million paired-end reads per library. In total, 87.67–88.65% of the reads were mapped to the *G. lucidum* genome sequence (Table S2). In this study, 10,938 genes were detected (Table S3). Comparison of the gene expression profiles in 3-octanone–treated and control fungi identified 590 DEGs (Fig. 1), including 162 upregulated and 428 downregulated genes.

### Functional annotation of DEGs

**Fig. 2:**
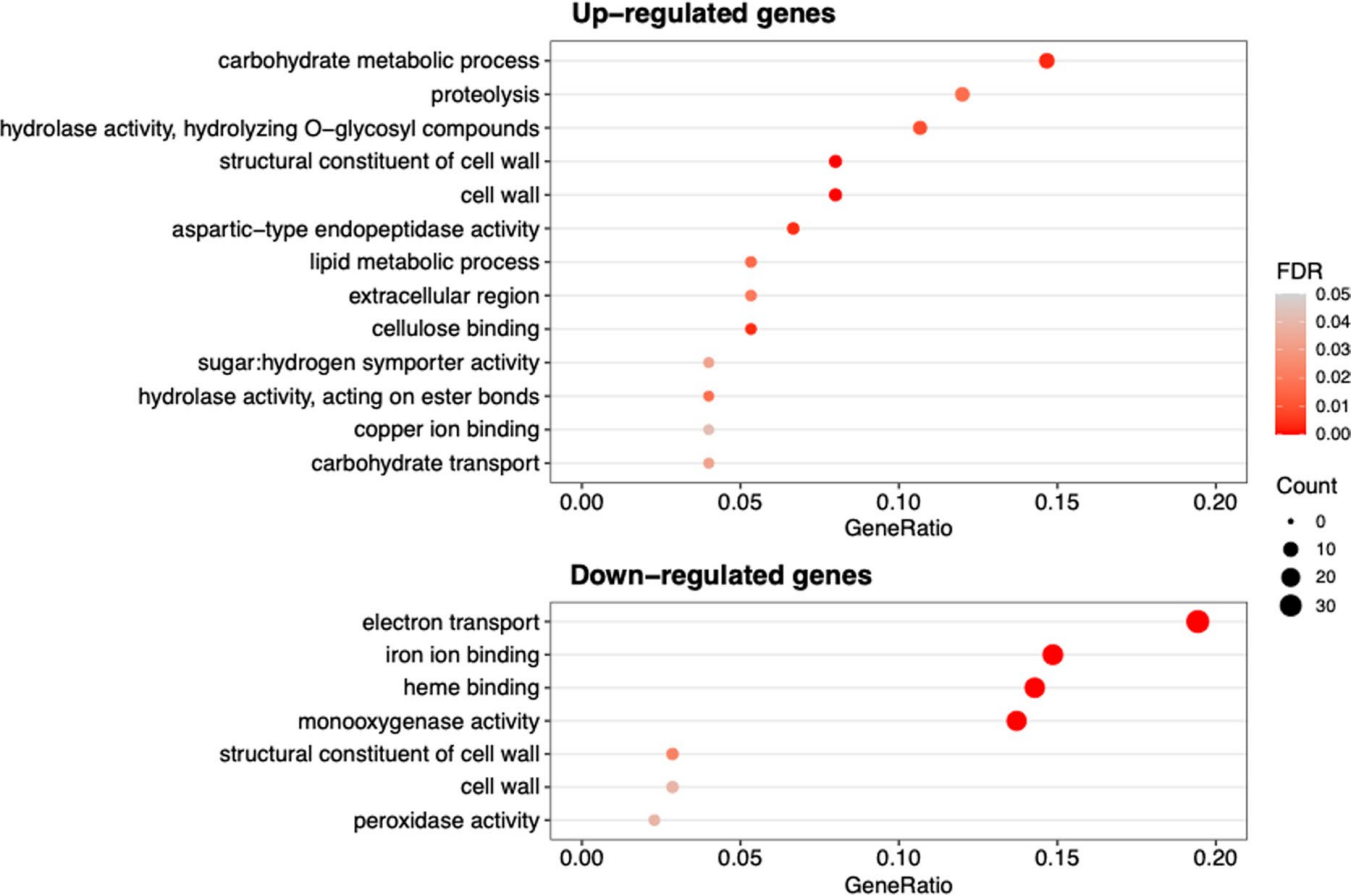
GO enrichment analysis of differentially expressed genes in *G. lucidum* upon 3-octanone treatment. Significantly enriched GO terms are shown for upregulated (upper) and downregulated (lower) genes. The x-axis represents the gene ratio, bar color indicates the FDR, and dot size corresponds to the number of genes in each GO term

GO enrichment analysis was performed to obtain insights into the functions of DEGs identified in *G. lucidum* following 3-octanone treatment. Thirteen GO terms were enriched in upregulated genes, whereas seven GO terms were enriched in downregulated genes (Fig. 2). Concerning upregulated genes, the most enriched GO term was “carbohydrate metabolic process,” followed by “proteolysis,” “hydrolase activity,” and “hydrolyzing O-glycosyl compounds,” suggesting significant changes in pathways related to carbon source metabolism. Furthermore, the GO terms “structural constituent of cell wall” and “cell wall” were enriched in both up- and downregulated genes, suggesting significant changes in cell wall structure. Among the downregulated genes, GO terms related to metal transport and binding, such as “electron transport,” “iron ion binding,” and “heme binding,” were notably enriched.

Meanwhile, 74 of the 590 DEGs were annotated as putative CAZymes, comprising annotations for 37 glycoside hydrolases (GHs), 13 glycosyltransferases (GTs), 6 carbohydrate esterases (CEs), and 14 auxiliary activity proteins (AAs), as well as nine genes containing carbohydrate-binding modules (CBMs) (Table S3). The fungal cell wall is a multilayer structure of polysaccharides, featuring a glucan network composed of *β*-1,3- and *β*-1,6-glucans surrounding chitin (Gow et al. 2017a, b). Furthermore, hydrophobins form a layer covering the cell wall polysaccharides (Ball et al. 2020). In the basidiomycete *Pleurotus ostreatus*, which possesses 40 hydrophobins in its genome and expresses them in a stage-specific manner, 11 were shown to be involved in mycelial growth and aerial mycelium formation (Xu et al. 2021). Of the 590 identified DEGs, 23 genes (3.9%) were suggested to have functions related to the fungal cell wall (Table 2). Among these, 11 genes were annotated as hydrophobins. Additionally, enzymes involved in hyphal growth, such as β-1,6-glucanase and chitinase, were identified among the DEGs. These included genes encoding members of the GH5, GH16, GH17, GH18, and GH152 families, which are known to be primarily associated with hyphal growth (Sakamoto et al. 2017). This upregulation of cell wall-related genes suggests that 3-octanone affects both the synthesis and remodeling of fungal cell walls.

**Table 2:** The up- and downregulated genes encoding fungal cell wall-related proteins in *G. lucidum* upon 3-octanone treatment

gene_id	logFC	CAZy family	Putative function
Up-regulated genes			
gene_77	1.93		Hydrophobin
gene_10695	1.58		Hydrophobin
gene_9681	1.58		Hydrophobin
gene_9491	1.44	GH18	Glycoside hydrolase, family 18
gene_10073	1.43	GH152	Thaumatin
gene_5909	1.29	GH152	Thaumatin
gene_7760	1.24		Hydrophobin
gene_10715	1.21		Hydrophobin
gene_10356	1.15		Hydrophobin
Down-regulated genes			
gene_13079	− 1.02	GH18	Glycoside hydrolase, chitinase active site
gene_6901	− 1.08	GH17	Glycoside hydrolase superfamily
gene_10204	− 1.11	GH5_12	Glutamine synthetase, catalytic domain
gene_1348	− 1.28	GH5_9	Glycoside hydrolase, family 5
gene_12255	− 1.67	GH16_4	Carboxylesterase
gene_1850	− 1.70		Hydrophobin
gene_10362	− 1.71		Hydrophobin
gene_10359	− 1.80		Hydrophobin
gene_13043	− 2.43		Hydrophobin
gene_3847	− 2.83	GH16_1	Concanavalin A-like lectin/glucanase domain
gene_10077	− 3.80	GH152	Thaumatin
gene_10358	− 3.83		Hydrophobin
gene_11488	− 3.86	GH16_4	Glycoside hydrolase, family 16
gene_11491	− 4.02	GH16_4	Glycoside hydrolase, family 16

^*^Positive logFC values denote upregulation, whereas negative values denote downregulation

In addition, 21 DEGs were postulated to participate in plant-derived polysaccharide degradation (Table 3). The most upregulated gene was annotated as an expansin-like protein belonging to the GH45 family. Furthermore, many genes involved in plant-derived polysaccharide degradation, including arabinosidase, which participates in hemicellulose side chains degradation, were found to be upregulated. These results suggest that 3-octanone enhances mycelial growth by activating the plant-derived polysaccharide degradation system.

**Table 3:** The up- and downregulated genes encoding proteins involved in plant-derived polysaccharide degradation in *G. lucidum* following 3-octanone treatment

gene_id	logFC	CAZy_Family	Putative function
*Up-regulated genes*			
gene_11273	3.79	GH45_3	Expansin
gene_13968	2.34	CBM1 | CE1	Cellulose-binding domain, fungal | Esterase, PHB depolymerase
gene_13306	1.99	CBM1 | GH5_5	Glycoside hydrolase, family 5 | Cellulose-binding domain, fungal
gene_12704	1.67	CE16	GDSL lipase/esterase
gene_4157	1.52	GH79	Glycoside hydrolase superfamily
gene_12713	1.46	CE16	GDSL lipase/esterase
gene_10745	1.42	GH31_1	Glycoside hydrolase family 31 |
gene_10071	1.40	GH43_6	Glycoside hydrolase, family 43, endo-1, 5-alpha-l-arabinosidase
gene_13587	1.35	AA1_1	Multicopper oxidase
gene_3821	1.22	CE12	SGNH hydrolase-type esterase
gene_13040	1.16	GH43_6	Glycoside hydrolase, family 43, endo-1, 5-alpha-l-arabinosidase
gene_8346	1.12	GH10	
gene_11726	1.08	GH28	Glycoside hydrolase, family 28
gene_11157	1.05	AA1_1	Multicopper oxidase
gene_6027	1.01	CBM1 | GH5_7	Glycoside hydrolase, family 5 | Cellulose-binding domain, fungal
*Down-regulated genes*			
gene_13636	− 1.11	GH13_32	Glycosyl hydrolase, family 13, alpha-amylase
gene_4093	− 1.25	CE4	Polysaccharide deacetylase
gene_3114	− 1.36	AA7	FAD-linked oxidase
gene_8116	− 1.41	GH13_1	Glycosyl hydrolase, family 13
gene_3886	− 1.63	GH79	Glycoside hydrolase
gene_3185	− 1.80	AA7	FAD-linked oxidase

^*^Positive logFC values denote upregulation, whereas negative values denote downregulation

In addition to genes involved in polysaccharide degradation, the DEGs also included genes involved in secondary metabolism (Table 4). The upregulated genes included some terpene synthases and a polyketide synthase, whereas the downregulated genes included trichodiene synthases and other terpene synthases. These results suggest that exposure to 3-octanone affects secondary metabolism in *G. lucidum*, particularly the volatile compound production.

**Table 4:** The up- and downregulated genes encoding proteins involved in secondary metabolism in *G. lucidum* following 3-octanone treatment

gene_id	logFC	Putative function
*Up-regulated genes*		
gene_3647	2.29	Terpene synthase
gene_12614	1.69	Terpene synthase
gene_8523	1.12	Ganoderic acid synthetase
gene_8347	1.06	Polyketide synthase
*Down-regulated genes*		
gene_5189	− 3.38	Trichodiene synthase
gene_3700	− 3.05	Trichodiene synthase
gene_7262	− 2.84	Terpene synthase
gene_3129	− 2.13	Terpene synthase
gene_3711	− 1.46	Trichodiene synthase
gene_3695	− 1.44	Trichodiene synthase
gene_3699	− 1.41	Trichodiene synthase
gene_13843	− 1.06	Trichodiene synthase

^*^Positive logFC values denote upregulation, whereas negative values denote downregulation

Based on our findings, we propose that 3-octanone, which has been detected during the cultivation of several wood-decaying fungi on wood (Konuma et al. 2015), promotes mycelial growth by acting as a signaling molecule that modulates fungal metabolism. This type of growth promotion by FVOCs was previously described by Evans et al. (2008), who found that the growth of a fungus in monoculture was promoted when it shared the same airspace with another plate in which two different fungal species were co-cultured in direct hyphal contact. This could represent a survival strategy of wood-rotting fungi, in which they sense environmental FVOCs as cues from neighboring fungi and adjust their metabolic pathways to expand their territory in nature.

Although the amount of 3-octanone supplied in this study is unlikely to contribute directly to fungal growth as a carbon source, alternative indirect mechanisms should be considered. In particular, exposure to volatile compounds may induce stress priming or modulate intracellular redox states, which could influence metabolic activity and growth-related processes. In the present study, our data do not allow us to distinguish between direct signal transduction and indirect metabolic activation mediated by stress or redox responses. Further experiments targeting redox-sensitive pathways, stress-response markers, and energy metabolism will be required to clarify the cellular processes linking FVOCs perception to mycelial growth promotion.

## Conclusions

In this study, we investigated changes in mycelial growth in the wood-rotting fungus *G. lucidum* following exposure to three FVOCs. On 1/4 diluted PDA medium, 3-octanone significantly promoted mycelial growth. RNA-seq analysis of 3-octanone–treated mycelia revealed that the expression of genes involved in fungal cell wall synthesis, plant-derived polysaccharide degradation, and secondary metabolism was significantly changed. These findings suggest that 3-octanone may act as a signaling molecule that regulates these biological processes. Although previous studies have highlighted the inhibitory effects of FVOCs in fungal interactions, our findings demonstrate that FVOCs can also promote growth by acting as signaling molecules, even without direct contact. These findings provide new insights into how wood-rotting fungi respond to volatile signals in their environment, which may be relevant to understanding fungal competition and niche expansion in forest ecosystems. In addition, the growth-promoting effects of specific FVOCs could be exploited to optimize cultivation strategies for medicinal and industrial fungi under low-nutrient conditions.

## Supplementary Information

Below is the link to the electronic supplementary material.

Supplementary file1 (XLSX 2342 KB)

## Data Availability

The datasets generated during and/or analyzed during the current study are available from the corresponding author on reasonable request.
